# Vascular Complication in Adolescents With Diabetes Mellitus

**DOI:** 10.3389/fendo.2020.00370

**Published:** 2020-06-09

**Authors:** Lara E. Graves, Kim C. Donaghue

**Affiliations:** ^1^Institute of Endocrinology and Diabetes, The Children's Hospital at Westmead, Westmead, NSW, Australia; ^2^Discipline of Child and Adolescent Health, Children's Hospital at Westmead Clinical School, University of Sydney, Westmead, NSW, Australia

**Keywords:** diabetes complications, microvascular, macrovascular, nephropathy, neuropathy, retinopathy

## Abstract

Diabetes mellitus is becoming more prevalent and even with new advancements which improve glycaemic control, complications of diabetes are common. Vascular complications of diabetes include the microvascular complications: retinopathy, nephropathy, and peripheral and autonomic neuropathy. Macrovascular complications are also common in patients with diabetes and arguably more concerning as they confer a high mortality risk yet are sometimes under-treated. Risk factors for diabetes complications start to occur in childhood and adolescents and some youths may be diagnosed with complications before transition to adult care. This article discusses the prevalence, risk factors, screening, and treatment recommendations for vascular complications in children and adolescents with diabetes.

## Introduction

Type 1 diabetes is becoming more prevalent and subclinical diabetes complications are common (1–3). While new advancements have improved glycaemic control and variability and reduced hypoglycaemic unawareness (4), long term complications are still an ongoing burden for patients with diabetes and 1 in 3 youths with type 1 diabetes have at least one diabetes complication (3). Microvascular complications are specific to diabetes and include retinopathy, peripheral neuropathy, autonomic neuropathy and nephropathy (5). The cells in the retina, nerves and renal glomeruli are unable to down-regulate glucose uptake, and in the presence of hyperglycaemia, this leads to an overproduction of superoxide in the mitochondria resulting in oxidative stress (5). Macrovascular complications are not specific to diabetes; however people with diabetes have accelerated atherosclerosis and a higher risk of macrovascular disease than the general population (5). More recently, microvascular complications appear to be decreasing in prevalence when compared with historical data (6), perhaps due in part to the advent of modern diabetes technology. Despite this, some risk factors for macrovascular disease such as obesity and hypertension are increasing (6) and there is a high rate of markers of inflammation associated with atherosclerosis in youth with diabetes (7). In this article we discuss the prevalence, risk factors, screening and treatment recommendations for vascular complications in children with type 1 diabetes. While this article focuses on type 1 diabetes, type 2 diabetes will also be discussed briefly as the prevalence of type 2 diabetes is also increasing in young people, and complications develop at a younger age (8), and may comprise a far greater proportion of the pediatric diabetes clinic in the future.

## The Vascular Complications of Diabetes

### Retinopathy

**Figure 1:**
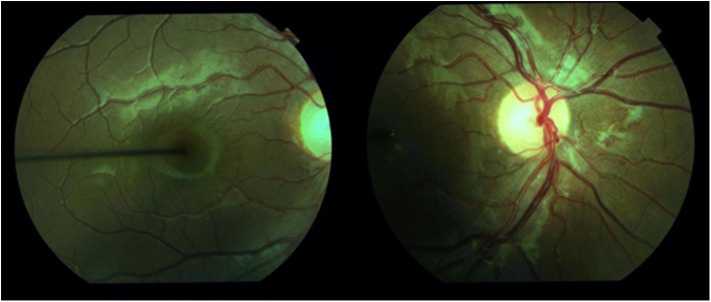
Retinal photography demonstrating proliferative retinopathy in a 15-year-old girl with 12 years diabetes duration and poor metabolic control. This had rapidly progressed from minimal retinopathy documented 2 years before.

Diabetes is the leading cause of new cases of blindness among adults aged 18–64 years (9). Diabetic eye disease encompasses diabetic retinopathy, macular oedema, cataract and glaucoma (5). Diabetic retinopathy is a microvascular complication of diabetes and is classified as mild-to-moderate non-proliferative, severe non-proliferative and proliferative. Severe non-proliferative and proliferative disease is vision threatening (10). Mild-to-moderate non-proliferative retinopathy is characterized by microaneurysms, retinal hemorrhages, ischemia, and microinfarction (cotton wool spots), protein and lipid leakage (hard exudates), intraretinal microvascular abnormalities and venular dilation and tortuosity. When there is vascular obstruction, increased number of retinal hemorrhages and microaneurysms with marked venous abnormalities, this is considered severe non-proliferative retinopathy. Proliferative retinopathy is characterized by neovascularisation in either the retina or posterior vitreous space (10). Progression of disease may be asymptomatic, as demonstrated in the case report (Figure 1). The presence of hard exudates with microaneurysms and blot hemorrhages within one disc diameter of the centre of the macular defines diabetic macular oedema (11). Clinically significant macular oedema has been traditionally defined as retinal thickening or hard exudate that involves or is within 500 μm of the fovea (12).

### Neuropathy

The most common type of diabetic neuropathy is peripheral neuropathy (diabetic sensorimotor polyneuropathy) but may also involve other parts of the somatic and autonomic nervous systems. In peripheral neuropathy, the sensory function is affected first followed by motor impairment. Patients may feel numbness and this may progress to pain (13).

Autonomic neuropathy may affect many systems including cardiovascular, urogenital or gastrointestinal and may present as orthostatic hypotension, exercise intolerance, resting tachycardia or bradycardia, reduced heart rate variability, gastroparesis, diarrhea, fecal or urinary incontinence, constipation, bladder paresis, and erectile dysfunction (14, 15). Loss of heart rate variability due to cardiovascular autonomic neuropathy may increase the risk of severe hypoglycaemia due to impairment of hypoglycaemia awareness (16). Cardiovascular manifestations can indicate sympathetic overdrive and loss of parasympathetic tone (16).

### Nephropathy

Diabetic nephropathy occurs due to interactions between multiple factors including increased systemic and intra-glomerular pressure, activation of the renin-angiotensin aldosterone system, activation of vascular endothelial growth factor, and from hyperglycaemia due to oxidative stress, renal polyol formation and accumulation of advanced glycation end products (17). This results in increasing proteinuria, glomerulosclerosis and eventually tubulointerstitial fibrosis (17). Albuminuria was formerly known as microalbuminuria. It is now generally defined based on the albumin-to-creatinine ratio. A ratio of 2.5–25 mg/mmol (30–300 mg/g) in males or a ratio of 3.5–25 mg/mmol (42–300 mg/g) in females defines albuminuria (10). Ratio levels above these ranges define proteinuria (10). The stages of diabetic nephropathy progress from renal hypertrophy, albuminuria (subclinical nephropathy), proteinuria (overt nephropathy), impairment of glomerular filtration rate (GFR) and finally to end-stage kidney disease (ESKD) (18, 19). Albuminuria is a risk factor for diabetic nephropathy, cardiovascular disease, cardiac autonomic dysfunction and mortality (5, 13, 20).

### Macrovascular

Macrovascular disease affects the coronary, cerebral and peripheral arterial vasculature. Patients with diabetes are at a higher risk of macrovascular disease than the general population and diabetes contributes to earlier mortality from cardiac disease. The duration of diabetes confers higher risk. The risk of coronary heart disease and acute myocardial infarction is 30 times increased in patients diagnosed with type 1 diabetes under the age of 10 years (21). Cardiac disease in type 1 diabetes is contributed to by accelerated atherosclerosis, cardiac autonomic neuropathy and intrinsic diabetic cardiomyopathy (22). Atherosclerosis acceleration is thought to be due to hyperglycaemia, glycation and oxidative stress causing endothelial dysfunction (22). It has been shown that youth with diabetes have higher levels of inflammatory biomarkers than their healthy peers, and that those with type 2 diabetes have higher inflammatory levels than those with type 1 diabetes (7).

Diabetic cardiomyopathy is when ventricular dysfunction occurs in patients with diabetes in the absence of coronary artery atherosclerosis and hypertension (22). Diabetic cardiomyopathy is caused by a combination of biochemical disturbances, impaired energy utilization, myocardial fibrosis, small vessel disease, cardiac autonomic neuropathy and insulin resistance (23). This can lead to diastolic and systolic dysfunction, which may present as shortness of breath or exercise intolerance (23). Diabetic cardiomyopathy may lead to diastolic heart failure (23). Otherwise healthy adolescents with type 1 diabetes have been shown to already have reduced exercise capacity and reduced stroke volume during exercise when compared to controls without diabetes (24). Functional cardiac changes occurred even after a relatively short disease duration mean of 6 years (24).

## Prevalence of Vascular Complications in Adolescents With Diabetes

Diabetes complications may develop in young people. Retinopathy is more common in young people with type 1 diabetes than type 2 diabetes, whereas albuminuria and hypertension are more common in young people with type 2 diabetes (25). In a group of 11–17 year old patients who had had type 1 diabetes for 2–5 years, early retinopathy was present in 9%, albuminuria was present in 3% and there was a peripheral nerve abnormality in 16% when measured by thermal and vibration thresholds (26). In our institution we had documented a reduction in retinopathy over two decades (1990–2009) in 12- to 20-year old patients with duration greater than five years (27), but this has plateaued since that report. Indeed, one female patient from our institute has subsequently required laser therapy before the age of 16 years due to proliferative retinopathy (Figure 1). In New Zealand between 2003 and 2016 there has been a decrease in microvascular complications with an increase in obesity and hypertension and no change in the prevalence of dyslipidaemia (6). However another New Zealand study found that in adolescents transitioning from pediatric to adult services between 2006 and 20016 there was no difference in the prevalence of albuminuria or hypertension over the 10 year period reported (28). A summary of the reported prevalence of diabetes complications in young people with type 1 diabetes is presented in Table 1. There is no standard definition of autonomic neuropathy and there is no recommended screening protocol, so the true prevalence is unclear (15).

**Table 1:** Epidemiology of complications of type 1 diabetes in children and adolescents.

**Complication**	**Study (number of participants)**	**Age (mean and/or range)**	**Diabetes duration**	**Mean HbA1c**	**Prevalence**
**RETINOPATHY**					
Grading 45° color digital fundus images centered on the disc and macula	SEARCH, (3) [1746]	17.9 years (mean), statistics adjusted for age 21 years	7.9 years	7.6%	5.6%
Stereoscopic fundal photography of seven fields	Cho et al. (26) [790]	11–17 years	2- to 5-years	8.5%	9%
Stereoscopic fundal photography of seven fields	Eppens et al. (25) [1264]	15.7 years (mean), 13.9–17.0 years	Age at diagnosis 8.1 (4.8–10.8) years	8.5%	20%
Retinal images, 2-fields included any retinopathy grade	Four Nations Diabetic Retinopathy Screening Group, (29) [2125]	12–13 years	Age at diagnosis <2 years	HbA1c not reported	20%
Retinal images, 2-fields included any retinopathy grade	Four Nations Diabetic Retinopathy Screening Group, (29) [2125]	12–13 years	Age at diagnosis 2–10 years	HbA1c not reported	8%
Stereoscopic fundal photography of seven fields	Aulich et al. (7) [134]	15.8 years	8.1 years (5.4–10.5)	8.5%	16%
**NEUROPATHY**					
Michigan Neuropathy Screening Instrument examination score >2	SEARCH, (3) [1746]	17.9 years (mean), statistics adjusted for age 21 years	7.9 years	7.6%	8.5%
Michigan Neuropathy Screening Instrument examination score >2	SEARCH, (30) [329]	15.7 years (mean)	6.2 ± 0.9 years	8.83%	8.2%
Thermal and vibration thresholds (abnormal was <5% of the normal range of control)	Eppens et al. (25) [1376]	15.7 years (mean), 13.9–17.0 years	Age at diagnosis 8.1 (4.8–10.8) years	8.5%	27%
Thermal and vibration thresholds (abnormal was outside of the 95th centile for control group)	Cho et al. (26) [803]	11–17 years	2- to 5-years	8.5%	16%
Thermal and vibration thresholds (abnormal was outside of the 95th centile for control group)	Aulich et al. (7) [134]	15.8 years	8.1 years (5.4–10.5)	8.5%	5%
**AUTONOMIC NEUROPATHY**					
Cardiovascular autonomic neuropathy using heart rate variability	SEARCH, (3) [1746]	17.9 years (mean), statistics adjusted for age 21 years	7.9 years	7.6%	14.4%
Pupil size before and after light stimulus (abnormal was <5% of the normal range of control)	Eppens et al. (25) [928]	15.7 years (mean), 13.9–17.0 years	Age at diagnosis 8.1 (4.8–10.8) years	8.5%	61%
Cardiovascular autonomic neuropathy using heart rate variability	Aulich et al. (7) [134]	15.8 years	8.1 years (5.4–10.5)	8.5%	28%
**NEPHROPATHY**					
Albuminuria or eGFR ≤ 60 mL/min/1.73 m^2^	SEARCH, (3) [1746]	17.9 years (mean), statistics adjusted for age 21 years	7.9 years	7.6%	5.8%
Albumin excretion rate ≥ 20 μg/min in at least 2 of 3 samples from timed overnight urine collections	Cho et al. (26) [729]	11–17 years	2- to 5-years	8.5%	3%
Mean albumin excretion rate on three consecutive, timed, overnight urine collections with 2 out of 3 samples AER ≥20 ug/min	Eppens et al. (25) [1325]	15.7 years (mean), 13.9–17.0 years	Age at diagnosis 8.1 (4.8–10.8) years	8.5%	6.1%
Albuminuria on urine albumin-to-creatinine ratio on at least two consecutive early morning urine samples	Amin et al. (31) [527]	18–20 years	9.8 years	9.5% (without albuminuria) 11.1% (with persistent albuminuria)^*^	26%
Albuminuria on urine albumin-to-creatinine ratio on at least two of three consecutive early morning samples or timed 24 h urine	Hornung et al. (28) [500]	16.9 years (mean)	8.7 years (=/- 4.1)	9%	4.6%
Albuminuria on urine albumin-to-creatinine ratio on at least two of three consecutive early morning samples or timed 24 h urine	T1D Exchange Clinic Network, (32) [7549]	13.8 ± 3.5 years	6.5 ± 7 years	8.4%	4.3%
Albuminuria on urine albumin-to-creatinine ratio on at least two of three consecutive early morning samples or timed 24 h urine	Aulich et al. (7) [134]	15.8 years	8.1 years (5.4–10.5)	8.5%	1%
**HYPERTENSION**					
	SEARCH, (3) [1746]	17.9 years (mean), statistics adjusted for age 21 years	7.9 years	7.6%	10.1%
	Eppens et al. (25) [1393]	15.7 years (mean), 13.9–17.0 years	Age at diagnosis 8.1 (4.8–10.8) years	8.5%	16%
	Hornung, et al. (28) [500]	16.9 years (mean)	8.7 years (=/- 4.1)	9%	2%
	Aulich et al. (7) [134]	15.8 years	8.1 years (5.4–10.5)	8.5%	9%
**MACRO-VASCULAR**					
Pulse wave velocity (arterial stiffness)	SEARCH, (33) [298]	19.2 years (mean)	4.8 years	8.9%	Significant increase of 0.145 m/s/year in PWV
Arterial stiffness	SEARCH, (3) [1746]	17.9 years (mean), statistics adjusted for age 21 years	7.9 years	7.6%	11.6%
Abnormal non-fasting lipid profile	Aulich et al. (7) [134]	15.8 years	8.1 years (5.4–10.5)	8.5%	14%
**DIABETIC CARDIOMYOPATHY**					
Functional aerobic capacity and cardiac MRI	Gusso et al. (24) [53]	15.6 years (mean)	6 ± 4 years	8.68%	10% decreased maximal exercise capacity compared with healthy age-matched controls. Reduced stroke volume in patients with type 1 diabetes. Increased systolic function at rest but not during exercise

* *HbA1c for entire cohort not provided. HbA1c provided for subgroups*.

Atherosclerosis starts in childhood and subclinical cardiovascular disease may be present in youth within 10 years of diagnosis with type 1 diabetes (34). The leading cause of morbidity and mortality in adults with type 1 diabetes is cardiovascular disease (35–38). Those diagnosed with diabetes under the age of 10 years have increased loss of life years and the risk of coronary artery disease and acute myocardial infarction is 30 times increased compared with those diagnosed with diabetes between ages 26–30 years (21). The first cardiovascular events occurred in the third decade of life for patients diagnosed with type 1 or type 2 diabetes at a young age (38). In a retrospective cohort of patients diagnosed with diabetes aged 15–30 years, 6% had evidence of macrovascular disease (38). Increased arterial stiffness independently predicts all-cause and cardiovascular mortality in adults with type 1 diabetes (39). Pulse wave velocity (PWV) is a measure of arterial stiffness, and there was a significant increase in PWV in youth with type 1 diabetes over a 5 year period (33). Atherosclerosis is associated with inflammation and biomarkers inflammatory markers (40). A cohort of patients with diabetes with a mean age of 15.6 years, had higher levels of biomarkers of inflammation than their healthy peers, particularly the subgroup with type 2 diabetes, compared with type 1 diabetes, and the overall rates of obesity were higher than that of the general population (7).

Young-onset type 2 diabetes confers a higher risk phenotype with greater mortality, more diabetes complications and more cardiovascular risk factors. The prevalence of type 2 diabetes in youth is increasing (41–43) and it is expected that diabetes complications in youth with type 2 diabetes will become an increasing burden as type 2 diabetes is associated with a higher rate of diabetes complications (43). Youth who have been diagnosed with type 2 diabetes for 1.8 years have similar rates of diabetes complications as youth who have had type 1 diabetes for 8.1 years (7). Youth with type 2 diabetes have a more rapid deterioration of beta cell function than the phenotype that develops in later adulthood (41). Obesity is an important contribution to insulin resistance seen in type 2 diabetes, and obesity itself is also associated with other metabolic risks (41). Complications of diabetes are often present at diagnosis of type 2. Within 2 years of diagnosis, the TODAY study found that adolescents aged 10–17 years already had evidence of diabetes complications or risk factors, including hypertension (13.6%), albuminuria (13%), low HDL level (79.8%), and hypertriglyceridemia (10.2%) (44). In this cohort there was a rapid rise in hypertension (12–34%) and albuminuria (6–17%) over the mean 4-year follow-up period (45). More recently, it was shown in a group of youth with type 2 diabetes with a mean age of 15.1 years and a mean duration of type 2 diabetes of 1.8 years 72% had evidence of any complication of diabetes. This included hypertension (19%), albuminuria (19%), heart rate variability abnormalities (54%), peripheral nerve abnormalities (19%), retinopathy (7%), and abnormal lipid profile (48%)(7). In a retrospective cohort of patients who had developed type 2 diabetes at a relatively young age (between ages 15 and 30 years), there is significant mortality excess when compared to patients who developed type 1 diabetes at a similar age: 11% mortality in patients with type 2 diabetes versus 6.8% mortality in patients with type 1 diabetes over median observation period >20 years, with more cardiovascular-attributable deaths in type 2 diabetes (50 vs. 30%) (38).

## Risk Factors for Diabetes Complications

Diabetes duration is the biggest risk factor for albuminuria and retinopathy (46) and is also a risk factor for peripheral neuropathy and macrovascular disease (13, 21). Other risk factors for micro and macrovascular disease include young age at diagnosis, higher HbA1c, higher blood pressure and socioeconomic disadvantage (13, 21, 27, 32, 47). There is a higher risk of progression of retinal disease in adolescents than in adults (48). The reason behind this is unclear but may be associated with the difficult in achieving glycaemic targets in this life period (49).

Lifestyle factors (such as smoking or poor exercise and diet), obesity, and dyslipidaemia are risk factors for peripheral neuropathy and macrovascular disease (13, 21). The presence of hypertension, diabetic kidney disease, insulin resistance or severe diabetic retinopathy are also risk factors for cardiovascular disease (21, 50). Furthermore depression (36) and hypoglycaemia (51) may also confer risk.

Youth with type 1 diabetes have a higher prevalence of dyslipidaemia (52), and there appears to be some sort of qualitative or functional abnormalities in lipoproteins in type 1 diabetes, which is more atherogenic (22). Dyslipidaemia is thought to be present in 48–80% of youth with type 2 diabetes within 2 years of diagnosis (7, 44).

Hypertension is a risk factor for microvascular complications and is also a major risk factor for macrovascular disease. Youth with type 1 diabetes have a higher prevalence of hypertension compared with their peers (53). For children aged 1–13 years, stage 1 hypertension is defined as “≥95th percentile to <95th percentile + 12 mmHg, or 130/80–139/89 mm Hg (whichever is lower)” and stage 2 hypertension is defined as “≥95th percentile + 12 mm Hg, or ≥140/90 mm Hg (whichever is lower)”. For children 13 years and over stage 1 is 130/80–139/89 mm Hg and stage 2 ≥140/90 mm Hg (54). It is confirmed by demonstration of elevated blood pressure on 3 separate occasions and may require 24 h ambulatory monitoring for confirmation (10, 55, 56). Normative data for blood pressure based on normal-weight children is available (54).

Obesity is a risk factor for both microvascular and macrovascular complications of diabetes. There are higher rates of overweight and obesity in youth with type 1 diabetes than their healthy peers (57–59), and BMI in youth may increase further overtime, particularly in girls (60). Albuminuria was associated with lower BMI in patients with type 1 diabetes under the age of 20 years (32). While causation was not examined, one might postulate that this could be due to poor weight gain due to relative insulin deficiency from under-dosing in patients with elevated HbA1c and poor adherence to therapy. Obese patients with diabetes have higher levels of inflammatory markers relative to other weight categories, and may have increased rates of diabetes complications (7).

Sustained hyperglycaemia is associated with diabetes complications and strict glycaemic control reduces both microvascular and macrovascular complications of diabetes (61, 62). It has been shown that an increasing HbA1c trajectory is associated with higher BMI, lower linear growth, use of insulin injection therapy and severe hypoglycaemic episodes (63), and poor glycaemic control early in life is associated with microvascular disease (64). Perhaps the HbA1c trajectory, not just the value, may also define risk of vascular complications and thus impact our approach.

The development and use of newer technology such as continuous glucose monitoring has provided detailed information about glucose handling in individual patients. The role of glycaemic variability in the development of complications is unclear (65, 66) and while it is recommended that the HbA1c target is <7% (67, 68), this may not be the only useful measure of complications risk. Thus both HbAc1c and “time in range” should be used together when devising treatment goals (69). Continuous subcutaneous insulin infusions using an insulin pump may reduce the risk of complications such as retinopathy and peripheral neuropathy (70). While it has been shown that the use of insulin pump therapy in some cohorts has been associated with improved glycaemic control (63, 71), diabetes technology has not necessarily been associated with a sustained improvement in glycaemic control or reduction in complications (72). Few studies have examined glucose variability using continuous glucose monitoring and diabetes complications as the widespread availability of continuous glucose monitoring is relatively recent. It is proposed that intermittent hyperglycaemia confers even greater oxidative stress than sustained hyperglycaemia, but long term studies looking at the glucose variability detected by continuous glucose monitoring and vascular complications are lacking (66, 73).

Microvascular disease has improved in a New Zealand cohort when compared with historical data, even in the absence of improvement in glycaemic control (6). Although this study did not address causation, perhaps reduction in glycaemic variability through continuous glucose monitoring has improved complications risk, without an obvious change to HbA1c. Despite an increase in the use of insulin pumps and continuous glucose monitoring in the US, the mean HbA1c in adolescents and young adults with type 1 diabetes has increased (72). Barriers to the use of diabetes technology in the United States may include non-English speaking background and ethnicity (74). Improving glycaemic control is not just related to the availability of technology but requires a multidisciplinary team approach which includes the patient and their family in management decisions (56). When a child and their parent manage the child's diabetes together, they are more likely to meet glycaemic targets (56).

When diabetes is diagnosed in childhood or adolescence, type 2 diabetes confers a higher risk for diabetic kidney disease, retinopathy and peripheral neuropathy than that for type 1 diabetes, but complications are frequent in both groups (3).

## Diabetes Complications Screening

The first signs of complications may appear during puberty and this is a critical time for the lifetime risk of diabetes (46). Risk factors should be considered at every visit (56) and at a minimum, blood pressure should be measured at least annually (10).

Regular screening for diabetic retinopathy has been recommended since the 1990s due to the asymptomatic progression to vision-threatening disease (61, 75–77). It is recommended that screening should commence from age 11 years after diabetes has been present for 2–5 years. Screening should be performed by an optometrist of ophthalmologist and should include a dilated and comprehensive eye examination. Current guidelines recommend eye screening should take place every 2 years in patients with good glycaemic control (10, 56), however more or less often may be appropriate depending on the individual clinical situation (78, 79).

Screening for albuminuria should commence from age 11 years after diabetes has been present for 2–5 years using three separate first morning urine samples. A positive result is considered if there is an abnormal urine albumin-to-creatinine ratio in 2 or more of the 3 samples. Screening should occur annually (10, 35, 56).

It is recommended that screening for peripheral neuropathy commences from age 11 years after diabetes has been present for 2–5 years and should be repeated annually (10, 56). Screening involves examining the foot with inspection and proprioceptive, vibratory and monofilament sensation should be assessed (56). Autonomic neuropathy is not generally screened for as there is no recommended screening technique available (10), but research methods can include measurement of heart rate for tachycardia, and reduced heart rate variability as a proxy for autonomic dysfunction (15).

Screening for dyslipidaemia should commence from 11 years of age, regardless of the duration of diabetes (10). This should not occur in the acute period shortly after diagnosis. Screening should commence at 2 years of age if there is family history significant for either hypercholesterolaemia or early cardiovascular death (10, 56). It is appropriate to screen with a non-fasting blood lipid profile, and if this is abnormal then a fasting profile should be performed (10, 56).

## Treatment Of Vascular Complications In Adolescents

### Retinopathy

Diabetic retinopathy may regress with improved glycaemic control but care must be taken with this as rapid improvement can actually lead to deterioration of retinopathy (10). The progression of retinopathy may be slowed with early use of angiotensin converting enzyme (ACE) inhibitors in adults, and this may be effective even when a patient does not have hypertension (80). However, this effect is yet to be demonstrated in adolescents (81).

Laser photocoagulation or antivascular endothelial growth factor (VEGF) intravitreal injections are required in the treatment of vision-threatening disease (10). Anti-VEGF may be preferred due to the risk of visual field reduction and night blindness associated with laser therapy (82). Anti-VEGF may be more effective prior to the development of proliferative disease (83), and hence early diagnosis is important. Fenofibrate has been used to reduce the need for laser treatment in adults with type 2 diabetes and the mechanism does not seem to be related to the effect on the plasma lipid concentration (84).

Surgery may be required if there is persistent vitreal hemorrhage or if lens extraction is required due to cataract (5, 10).

### Lifestyle Measures

The first steps in management of hypertension or dyslipidaemia, are lifestyle measures to optimize body habitus and exercise tolerance and improvement in glycaemic control (10, 85). The use of dietary adjuncts such as plant sterols could be considered in the presence of dyslipidaemia (86). If lifestyle measures including diet and exercise are optimized for 3–6 months and there is persistence of hypertension or dyslipidaemia, then pharmacotherapy should be considered (10, 54, 56). There is often therapeutic inertia to introduce further appropriate treatments in youth (36), but early intervention is imperative and treatment that is indicated should not be withheld.

### Anti-hypertensives

Hypertension and albuminuria may be considered surrogate markers of diabetic kidney disease. ACE inhibitors have been shown to decrease albuminuria in children even in the absence of hypertension and should be used to treat children with albuminuria to prevent progression to proteinuria (56, 87). With tight glycaemic control and the use of ACE inhibitors or angiotensin receptor blockers (ARB), albuminuria can improve (14). Hypertension may also be treated with ACE inhibitors, ARB, calcium channel blockers or thiazide diuretic (10, 54, 56), however if concomitant albuminuria is present, treatment with an ACE inhibitor or ARB is recommended. There is no specific recommendation for type of ACE inhibitor and many are appropriate for pediatric use including benazepril, captopril, enalapril, fosinopril or lisinopril (54).

### Statins

Statins effectively treat dyslipidaemia in adolescents with type 1 diabetes (10, 56, 81). If the LDL cholesterol remains greater than 3.4 mmol/L despite lifestyle measures, statins should be considered from the age of 11 years. The use of statins is not approved for children under 10 years (56). Atorvastatin, lovastatin or pravastatin have been trialed in children and adolescents and shown to be safe and effective in the treatment of dyslipidaemia (81, 88–90). Dyslipidaemia that is being treated with pharmacotherapy should be monitored with fasted lipid profiles, targeting a fasting LDL cholesterol of <2.6 mmol/L (56).

### Smoking

In addition to management of hypertension and dyslipidaemia, young people should be counseled to avoid or cease smoking cigarettes, as smoking avoidance helps prevent both microvascular and macrovascular complications (56).

### Metformin

Metformin is recommended in the treatment of type 2 diabetes in youth (91). It may also help reduce the BMI and insulin requirements in overweight youth with type 1 diabetes (92, 93). Metformin may have an additional role in the cardiovascular health of patients with diabetes. Metformin was shown to improve vascular smooth muscle function independently of any improvement in glycaemic control and insulin sensitivity in adolescents with type 1 diabetes (94). In patients aged 12 and 21 years with type 1 diabetes metformin use showed improvement in MRI-derived measures of aortic and carotid vascular health (93).

## Conclusion

Vascular complications of diabetes are common in youth with diabetes, and infer a high morbidity and mortality risk (2, 3). Prevention is critical, by targeting risk factors such as glycaemic control, adiposity, hypertension, dyslipidaemia and life style factors. Improvements may be achieved using appropriate diabetes technology, a multidisciplinary team approach and health education (61, 62, 65). Screening for complications is crucial as at-risk adolescents must be identified and treatment commenced before irreversible changes occur.

## Author Contributions

LG and KD researched the scientific literature, wrote the review and edited the review before submission. LG prepared the tables.

## Conflict of Interest

KD receives research support from the Australian National Health and Medical Research Council and Diabetes Australia, and her institution has received research support from JDRF and Medtronic. She has received speaker fees from Eli Lilly. The remaining author declares that the research was conducted in the absence of any commercial or financial relationships that could be construed as a potential conflict of interest.

## References

[1] Dabelea D. Diabetes in youth-looking backwards to inform the future: Kelly West Award Lecture 2017. Diabetes Care. (2018) 41:233–40. 10.2337/dci17-0031 29358467 PMC5780050

[2] Hamman RF Bell RA Dabelea D D'Agostino RB Jr Dolan L. The SEARCH for diabetes in youth study: Rationale, findings, and future directions. Diabetes Care. (2014) 37:3336–44.d 10.2337/dc14-0574 25414389 PMC4237981

[3] Dabelea D Stafford JM Mayer-Davis EJ D'Agostino R Jr Dolan L. Association of type 1 diabetes vs. type 2 diabetes diagnosed during childhood and adolescence with complications during teenage years and young adulthood. JAMA. (2017) 317:825–35. 10.1001/jama.2017.0686 28245334 PMC5483855

[4] O'Connell PJ Hawthorne WJ Holmes-Walker DJ Nankivell BJ Gunton JE Patel AT. Clinical islet transplantation in type 1 diabetes mellitus: Results of Australia's first trial. Med J Aust. (2006) 184:221–5. 10.5694/j.1326-5377.2006.tb00206.x 16515432

[5] Katsarou A Gudbjornsdottir S Rawshani A Dabelea D Bonifacio E Anderson BJ. Type 1 diabetes mellitus. Nat Rev Dis Primers. (2017) 3:17016. 10.1038/nrdp.2017.16 28358037

[6] Sandhu SK Corbett VM Chepulis L Goldsmith J Joseph P Fraser SK. The prevalence of microvascular complications in Waikato children and youth with type 1 diabetes has reduced since 2003. N Z Med J. (2020) 133:35–44. 32078599

[7] Aulich J Cho YH Januszewski AS Craig ME Selvadurai H Wiegand S. Associations between circulating inflammatory markers, diabetes type and complications in youth. Pediatr Diabetes. (2019) 20:1118–27. 10.1111/pedi.12913 31464058

[8] Dart AB Martens PJ Rigatto C Brownell MD Dean HJ Sellers EA. Earlier onset of complications in youth with type 2 diabetes. Diabetes Care. (2014) 37:436–43. 10.2337/dc13-0954 24130346

[9] Centers for Disease Control Prevention National Diabetes Statistics Report, 2020. Atlanta, GA: Centers for Disease Control Prevention, US Dept of Health and Human Services. (2020). Available online at: https://www.cdc.gov/diabetes/pdfs/data/statistics/national-diabetes-statistics-report.pdf.

[10] Donaghue KC Marcovecchio ML Wadwa RP Chew EY Wong TY Calliari LE. ISPAD Clinical Practice Consensus Guidelines 2018: Microvascular and macrovascular complications in children and adolescents. Pediatr Diabetes. (2018) 19 Suppl 27:262–74. 10.1111/pedi.12742 30079595 PMC8559793

[11] Lee R Wong TY Sabanayagam C. Epidemiology of diabetic retinopathy, diabetic macular edema and related vision loss. Eye Vis (Lond). (2015) 2:17. 10.1186/s40662-015-0026-2 26605370 PMC4657234

[12] Early Treatment Diabetic Retinopathy Study design and baseline patient characteristics ETDRS report number 7. Ophthalmology. (1991). 98(5 Suppl):741–56. 10.1016/S0161-6420(13)38009-9 2062510

[13] Jaiswal M Divers J Dabelea D Isom S Bell RA Martin CL. Prevalence of and risk factors for diabetic peripheral neuropathy in youth with type 1 and type 2 diabetes: SEARCH for diabetes in youth study. Diabetes Care. (2017) 40:1226–32. 10.2337/dc17-0179 28674076 PMC5566278

[14] Perkins BA Ficociello LH Silva KH Finkelstein DM Warram JH Krolewski AS. Regression of microalbuminuria in type 1 diabetes. N Engl J Med. (2003) 348:2285–93. 10.1056/NEJMoa021835 12788992

[15] Tang M Donaghue KC Cho YH Craig ME. Autonomic neuropathy in young people with type 1 diabetes: a systematic review. Pediatr Diabetes. (2013) 14:239–48. 10.1111/pedi.12039 23627912

[16] da Silva T Rolim L de Camargo Sallum Filho C Zimmermann LM Malerbi F Dib S. Impaired awareness of hypoglycemia is associated with progressive loss of heart rate variability in patients with type 1 diabetes. Diabetol Metabol Syndr. (2015) 7(Suppl 1):A63. 10.1186/1758-5996-7-S1-A6327239809

[17] Soldatos G Cooper ME. Diabetic nephropathy: important pathophysiologic mechanisms. Diabetes Res Clin Pract. (2008) 82 Suppl 1:S75–9. 10.1016/j.diabres.2008.09.042 18994672

[18] Mogensen CE. Microalbuminuria in prediction and prevention of diabetic nephropathy in insulin-dependent diabetes mellitus patients. J Diabetes Compl. (1995) 9:337–49. 10.1016/1056-8727(95)80036-E 8573761

[19] Mogensen CE. Drug treatment for hypertensive patients in special situations: diabetes and hypertension. Clin Exp Hypertens. (1999) 21:895–906. 10.3109/10641969909061018 10423111

[20] Rossing P Hougaard P Borch-Johnsen K Parving HH. Predictors of mortality in insulin dependent diabetes: 10 year observational follow up study. BMJ. (1996) 313:779–84. 10.1136/bmj.313.7060.779 8842069 PMC2352213

[21] Rawshani A Sattar N Franzen S Rawshani A Hattersley AT Svensson AM. Excess mortality and cardiovascular disease in young adults with type 1 diabetes in relation to age at onset: a nationwide, register-based cohort study. Lancet. (2018) 392:477–86. 10.1016/S0140-6736(18)31506-X 30129464 PMC6828554

[22] Retnakaran R Zinman B. Type 1 diabetes, hyperglycaemia, and the heart. Lancet. (2008) 371:1790–9. 10.1016/S0140-6736(08)60767-9 18502304

[23] Fang ZY Prins JB Marwick TH. Diabetic cardiomyopathy: evidence, mechanisms, and therapeutic implications. Endocr Rev. (2004) 25:543–67. 10.1210/er.2003-0012 15294881

[24] Gusso S Pinto TE Baldi JC Robinson E Cutfield WS Hofman PL. Diastolic function is reduced in adolescents with type 1 diabetes in response to exercise. Diabetes Care. (2012) 35:2089–94. 10.2337/dc11-2331 22773700 PMC3447841

[25] Eppens MC Craig ME Cusumano J Hing S Chan AK Howard NJ. Prevalence of diabetes complications in adolescents with type 2 compared with type 1 diabetes. Diabetes Care. (2006) 29:1300–6. 10.2337/dc05-2470 16732012

[26] Cho YH Craig ME Hing S Gallego PH Poon M Chan A. Microvascular complications assessment in adolescents with 2- to 5-yr duration of type 1 diabetes from 1990 to (2006). Pediatr Diabetes. (2011) 12:682–9. 10.1111/j.1399-5448.2011.00762.x 21435138

[27] Downie E Craig ME Hing S Cusumano J Chan AK Donaghue KC. Continued reduction in the prevalence of retinopathy in adolescents with type 1 diabetes: role of insulin therapy and glycemic control. Diabetes Care. (2011) 34:2368–73. 10.2337/dc11-0102 22025782 PMC3198305

[28] Hornung RJ Reed PW Mouat F Jefferies C Gunn AJ Hofman PL. Angiotensin-converting enzyme-inhibitor therapy in adolescents with type 1 diabetes in a regional cohort: Auckland, New Zealand from 2006 to 2016. J Paediatr Child Health. (2018) 54:493–8. 10.1111/jpc.13814 29271523

[29] Scanlon PH Stratton IM Bachmann MO Jones C Leese GP Four Nations Diabetic Retinopathy Screening Study G. Risk of diabetic retinopathy at first screen in children at 12 and 13 years of age. Diabet Med. (2016) 33:1655–8. 10.1111/dme.13263 27646856 PMC5434868

[30] Jaiswal M Lauer A Martin CL Bell RA Divers J Dabelea D Peripheral neuropathy in adolescents and young adults with type 1 and type 2 diabetes from the SEARCH for Diabetes in Youth follow-up cohort: a pilot study. Diabetes Care. (2013) 36:3903–8. 10.2337/dc13-1213 24144652 PMC3836139

[31] Amin R Widmer B Prevost AT Schwarze P Cooper J Edge J. Risk of microalbuminuria and progression to macroalbuminuria in a cohort with childhood onset type 1 diabetes: prospective observational study. British Medical Journal. (2008) 336:697–701. 10.1136/bmj.39478.378241.BE 18349042 PMC2276285

[32] Daniels M DuBose SN Maahs DM Beck RW Fox LA Gubitosi-Klug R. Factors associated with microalbuminuria in 7,549 children and adolescents with type 1 diabetes in the T1D Exchange clinic registry. Diabetes Care. (2013) 36:2639–45. 10.2337/dc12-2192 23610082 PMC3747908

[33] Dabelea D Talton JW D'Agostino R Jr Wadwa RP Urbina EM Cardiovascular risk factors are associated with increased arterial stiffness in youth with type 1 diabetes: the SEARCH CVD study. Diabetes Care. (2013) 36:3938–43. 10.2337/dc13-0851 24101697 PMC3836140

[34] Singh TP Groehn H Kazmers A. Vascular function and carotid intimal-medial thickness in children with insulin-dependent diabetes mellitus. J Am Coll Cardiol. (2003) 41:661–5. 10.1016/S0735-1097(02)02894-2 12598080

[35] Chiang JL Maahs DM Garvey KC Hood KK Laffel LM Weinzimer SA. Type 1 diabetes in children and adolescents: a position statement by the American Diabetes Association. Diabetes Care. (2018) 41:2026–44. 10.2337/dci18-0023 30093549 PMC6105320

[36] Bjornstad P Donaghue KC Maahs DM. Macrovascular disease and risk factors in youth with type 1 diabetes: time to be more attentive to treatment? Lancet Diabetes Endocrinol. (2018) 6:809–20. 10.1016/S2213-8587(18)30035-4 29475800 PMC6102087

[37] Krolewski AS Kosinski EJ Warram JH Leland OS Busick EJ Asmal AC. Magnitude and determinants of coronary artery disease in juvenile-onset, insulin-dependent diabetes mellitus. Am J Cardiol. (1987) 59:750–5. 10.1016/0002-9149(87)91086-1 3825934

[38] Constantino MI Molyneaux L Limacher-Gisler F Al-Saeed A Luo C Wu T. Long-term complications and mortality in young-onset diabetes: type 2 diabetes is more hazardous and lethal than type 1 diabetes. Diabetes Care. (2013) 36:3863–9. 10.2337/dc12-2455 23846814 PMC3836093

[39] Cruickshank K Riste L Anderson SG Wright JS Dunn G Gosling RG. Aortic pulse-wave velocity and its relationship to mortality in diabetes and glucose intolerance: an integrated index of vascular function? Circulation. (2002) 106:2085–90. 10.1161/01.CIR.0000033824.02722.F7 12379578

[40] Berliner JA Navab M Fogelman AM Frank JS Demer LL Edwards PA. Atherosclerosis: basic mechanisms. Oxidation, inflammation, and genetics. Circulation. (1995) 91:2488–96. 10.1161/01.CIR.91.9.2488 7729036

[41] D'Adamo E Caprio S. Type 2 diabetes in youth: epidemiology and pathophysiology. Diabetes Care. (2011) 34 Suppl 2:S161–5. 10.2337/dc11-s212 21525449 PMC3632155

[42] Pinhas-Hamiel O Zeitler P. The global spread of type 2 diabetes mellitus in children and adolescents. J Pediatr. (2005) 146:693–700. 10.1016/j.jpeds.2004.12.042 15870677

[43] Jensen ET Dabelea D. Type 2 Diabetes in Youth: New Lessons from the SEARCH Study. Curr Diab Rep. (2018) 18:36. 10.1007/s11892-018-0997-1 29737424 PMC6438166

[44] Copeland KC Zeitler P Geffner M Guandalini C Higgins J Hirst K. Characteristics of adolescents and youth with recent-onset type 2 diabetes: the TODAY cohort at baseline. J Clin Endocrinol Metab. (2011) 96:159–67. 10.1210/jc.2010-1642 20962021 PMC3038479

[45] Today Study Group. Rapid rise in hypertension and nephropathy in youth with type 2 diabetes: the TODAY clinical trial. Diabetes Care. (2013) 36:1735–41. 10.2337/dc12-2420 23704672 PMC3661847

[46] Dunger DB. Banting Memorial Lecture 2016. Reducing lifetime risk of complications in adolescents with type 1 diabetes. Diabet Med. (2017) 34:460–6. 10.1111/dme.13299 27973749

[47] Gallego PH Craig ME Hing S Donaghue KC. Role of blood pressure in development of early retinopathy in adolescents with type 1 diabetes: prospective cohort study. BMJ. (2008) 337:a918. 10.1136/bmj.a918 18728082 PMC2526183

[48] Hietala K Harjutsalo V Forsblom C Summanen P Groop PH FinnDiane Study G. Age at onset and the risk of proliferative retinopathy in type 1 diabetes. Diabetes Care. (2010) 33:1315–9. 10.2337/dc09-2278 20185730 PMC2875446

[49] White NH Cleary PA Dahms W Goldstein D Malone J Tamborlane WV. Beneficial effects of intensive therapy of diabetes during adolescence: outcomes after the conclusion of the Diabetes Control and Complications Trial (DCCT). J Pediatr. (2001) 139:804–12. 10.1067/mpd.2001.118887 11743505

[50] Pongrac Barlovic D Harjutsalo V Gordin D Kallio M Forsblom C King G. The association of severe diabetic retinopathy with cardiovascular outcomes in long-standing type 1 diabetes: a longitudinal follow-up. Diabetes Care. (2018) 41:2487–94. 10.2337/dc18-0476 30257963 PMC6973548

[51] Pena AS Couper JJ Harrington J Gent R Fairchild J Tham E. Hypoglycemia, but not glucose variability, relates to vascular function in children with type 1 diabetes. Diabetes Technol Ther. (2012) 14:457–62. 10.1089/dia.2011.0229 22313018 PMC3359626

[52] Schwab KO Doerfer J Marg W Schober E Holl RW Initiative DPVS. Characterization of 33 488 children and adolescents with type 1 diabetes based on the gender-specific increase of cardiovascular risk factors. Pediatr Diabetes. (2010) 11:357–63. 10.1111/j.1399-5448.2010.00665.x 20624248

[53] Knerr I Dost A Lepler R Raile K Schober E Rascher W. Tracking and prediction of arterial blood pressure from childhood to young adulthood in 868 patients with type 1 diabetes: a multicenter longitudinal survey in Germany and Austria. Diabetes Care. (2008) 31:726–7. 10.2337/dc07-1392 18184906

[54] Flynn JT Kaelber DC Baker-Smith CM Blowey D Carroll AE Daniels SR. Clinical practice guideline for screening and management of high blood pressure in children and adolescents. Pediatrics. (2017) 140:e20171904. 10.1542/peds.2017-3035 28827377

[55] Soergel M Kirschstein M Busch C Danne T Gellermann J Holl R. Oscillometric twenty-four-hour ambulatory blood pressure values in healthy children and adolescents: a multicenter trial including 1141 subjects. J Pediatr. (1997) 130:178–84. 10.1016/S0022-3476(97)70340-8 9042117

[56] American Diabetes A. 13. Children and adolescents: standards of medical care in diabetes-2019. Diabetes Care. (2019) 42(Suppl 1):S148-S64. 10.2337/dc19-S013 30559239

[57] DuBose SN Hermann JM Tamborlane WV Beck RW Dost A DiMeglio LA. Obesity in youth with type 1 diabetes in Germany, Austria, and the United States. J Pediatr. (2015) 167:627–32 e1–4. 10.1016/j.jpeds.2015.05.046 26164381

[58] Redondo MJ Foster NC Libman IM Mehta SN Hathway JM Bethin KE. Prevalence of cardiovascular risk factors in youth with type 1 diabetes and elevated body mass index. Acta Diabetol. (2016) 53:271–7. 10.1007/s00592-015-0785-1 26077171

[59] Phelan H Clapin H Bruns L Cameron FJ Cotterill AM Couper JJ. The Australasian Diabetes Data Network: first national audit of children and adolescents with type 1 diabetes. Med J Aust. (2017) 206:121–5. 10.5694/mja16.00737 28208043

[60] Phelan H Foster NC Schwandt A Couper JJ Willi S Kroschwald P. Longitudinal trajectories of BMI z-score: an international comparison of 11,513 Australian, American and German/Austrian/Luxembourgian youth with type 1 diabetes. Pediatr Obes. (2020) 15:e12582. 10.1111/ijpo.12582 31691541

[61] Nathan DM Genuth S Lachin J Cleary P Crofford O Davis M. The effect of intensive treatment of diabetes on the development and progression of long-term complications in insulin-dependent diabetes mellitus. N Engl J Med. (1993) 329:977–86. 10.1056/NEJM199309303291401 8366922

[62] Stratton IM Adler AI Neil HA Matthews DR Manley SE Cull CA. Association of glycaemia with macrovascular and microvascular complications of type 2 diabetes (UKPDS 35): prospective observational study. BMJ. (2000) 321:405–12. 10.1136/bmj.321.7258.405 10938048 PMC27454

[63] Clements MA Schwandt A Donaghue KC Miller K Luck U Couper JJ. Five heterogeneous HbA1c trajectories from childhood to adulthood in youth with type 1 diabetes from three different continents: a group-based modeling approach. Pediatr Diabetes. (2019) 20:920–31. 10.1111/pedi.12907 31418521

[64] Writing Team for the Diabetes C Complications Trial/Epidemiology of Diabetes I Complications Research G Effect of intensive therapy on the microvascular complications of type 1 diabetes mellitus. JAMA. (2002) 287:2563–9. 10.1001/jama.287.19.2563 12020338 PMC2622728

[65] Monnier L Colette C Owens D. The glycemic triumvirate and diabetic complications: is the whole greater than the sum of its component parts? Diabetes Res Clin Pract. (2012) 95:303–11. 10.1016/j.diabres.2011.10.014 22056719

[66] Ceriello A Monnier L Owens D. Glycaemic variability in diabetes: clinical and therapeutic implications. Lancet Diabetes Endocrinol. (2019) 7:221–30. 10.1016/S2213-8587(18)30136-0 30115599

[67] DiMeglio LA Acerini CL Codner E Craig ME Hofer SE Pillay K ISPAD Clinical Practice Consensus Guidelines 2018: glycemic control targets and glucose monitoring for children, adolescents, and young adults with diabetes. Pediatr Diabetes. (2018) 19 Suppl 27:105–14. 10.1111/pedi.12737 30058221

[68] The National Institute for Health and Care Excellence (NICE) Guideline 18: Diabetes (type 1 and type 2) in Children and Young People: Diagnosis and Management London (2015). Available online at: http://nice.org.uk/guidance/ng18.

[69] Danne T Nimri R Battelino T Bergenstal RM Close KL DeVries JH. International consensus on use of continuous glucose monitoring. Diabetes Care. (2017) 40:1631–40. 10.2337/dc17-1600 29162583 PMC6467165

[70] Zabeen B Craig ME Virk SA Pryke A Chan AK Cho YH. Insulin pump therapy is associated with lower rates of retinopathy and peripheral nerve abnormality. PLoS One. (2016) 11:e0153033. 10.1371/journal.pone.0153033 27050468 PMC4822832

[71] Mair C Wulaningsih W Jeyam A McGurnaghan S Blackbourn L Kennon B. Glycaemic control trends in people with type 1 diabetes in Scotland 2004-2016. Diabetologia. (2019) 62:1375–84. 10.1007/s00125-019-4900-7 31104095 PMC6647722

[72] Foster NC Beck RW Miller KM Clements MA Rickels MR DiMeglio LA. State of type 1 diabetes management and outcomes from the t1d exchange in 2016-2018. Diabetes Technol Ther. (2019) 21:66–72. 10.1089/dia.2018.0384 30657336 PMC7061293

[73] Jung HS. Clinical implications of glucose variability: chronic complications of diabetes. Endocrinol Metab (Seoul). (2015) 30:167–74. 10.3803/EnM.2015.30.2.167 26194076 PMC4508260

[74] O'Connor MR Carlin K Coker T Zierler B Pihoker C. Disparities in insulin pump therapy persist in youth with type 1 diabetes despite rising overall pump use rates. J Pediatr Nurs. (2019) 44:16–21. 10.1016/j.pedn.2018.10.005 30581163 PMC10602396

[75] Kernell A Dedorsson I Johansson B Wickstrom CP Ludvigsson J Tuvemo T. Prevalence of diabetic retinopathy in children and adolescents with IDDM. A population-based multicentre study. Diabetologia. (1997) 40:307–10. 10.1007/s001250050679 9084969

[76] Diabetes Control and Complications Trial Research Group Effect of intensive diabetes treatment on the development and progression of long-term complications in adolescents with insulin-dependent diabetes mellitus: diabetes control and complications trial. J Pediatr. (1994) 125:177–88. 10.1016/S0022-3476(94)70190-3 8040759

[77] Retinopathy Working Party. A protocol for screening for diabetic retinopathy in Europe. Diabet Med. (1991). 8:263–7. 10.1111/j.1464-5491.1991.tb01583.x 1828743

[78] Group DER Nathan DM Bebu I Hainsworth D Klein R Tamborlane W. Frequency of evidence-based screening for retinopathy in type 1 diabetes. N Engl J Med. (2017) 376:1507–16. 10.1056/NEJMoa1612836 28423305 PMC5557280

[79] Gubitosi-Klug RA Bebu I White NH Malone J Miller R Lorenzi GM. Screening eye exams in youth with type 1 diabetes under 18 years of age: once may be enough? Pediatr Diabetes. (2019) 20:743–9. 10.1111/pedi.12877 31206973 PMC7217664

[80] Mauer M Zinman B Gardiner R Suissa S Sinaiko A Strand T. Renal and retinal effects of enalapril and losartan in type 1 diabetes. N Engl J Med. (2009) 361:40–51. 10.1056/NEJMoa0808400 19571282 PMC2978030

[81] Marcovecchio ML Chiesa ST Bond S Daneman D Dawson S Donaghue KC. ACE inhibitors and statins in adolescents with type 1 diabetes. N Engl J Med. (2017) 377:1733–45. 10.1056/NEJMoa1703518 29091568

[82] Aiello LM. Perspectives on diabetic retinopathy. Am J Ophthalmol. (2003) 136:122–35. 10.1016/S0002-9394(03)00219-8 12834680

[83] Ip MS Domalpally A Sun JK Ehrlich JS. Long-term effects of therapy with ranibizumab on diabetic retinopathy severity and baseline risk factors for worsening retinopathy. Ophthalmology. (2015) 122:367–74. 10.1016/j.ophtha.2014.08.048 25439595

[84] Keech A Mitchell P Summanen P O'Day J Davis T Moffitt M. Effect of fenofibrate on the need for laser treatment for diabetic retinopathy (FIELD study): a randomised controlled trial. Lancet. (2007) 370:1687–97. 10.1016/S0140-6736(07)61607-9 17988728

[85] Maahs DM Daniels SR de Ferranti SD Dichek HL Flynn J Goldstein BI. Cardiovascular disease risk factors in youth with diabetes mellitus: a scientific statement from the American Heart Association. Circulation. (2014) 130:1532–58. 10.1161/CIR.0000000000000094 25170098

[86] Expert Panel on Integrated Guidelines for Cardiovascular H Risk Reduction in C Adolescents National Heart L Blood I Expert panel on integrated guidelines for cardiovascular health and risk reduction in children and adolescents: summary report. Pediatrics. (2011) 128 Suppl 5:S213–56. 10.1542/peds.2009-2107C 22084329 PMC4536582

[87] Cook J Daneman D Spino M Sochett E Perlman K Balfe JW. Angiotensin converting enzyme inhibitor therapy to decrease microalbuminuria in normotensive children with insulin-dependent diabetes mellitus. J Pediatr. (1990) 117(1 Pt 1):39–45. 10.1016/S0022-3476(05)82441-2 2196359

[88] Langslet G Breazna A Drogari E. A 3-year study of atorvastatin in children and adolescents with heterozygous familial hypercholesterolemia. J Clin Lipidol. (2016) 10:1153–62 e3. 10.1016/j.jacl.2016.05.010 27678432

[89] Stein EA Illingworth DR Kwiterovich PO Jr Liacouras CA Siimes MA. Efficacy and safety of lovastatin in adolescent males with heterozygous familial hypercholesterolemia: a randomized controlled trial. JAMA. (1999) 281:137–44. 10.1001/jama.281.2.137 9917116

[90] Wiegman A Hutten BA de Groot E Rodenburg J Bakker HD Buller HR. Efficacy and safety of statin therapy in children with familial hypercholesterolemia: a randomized controlled trial. JAMA. (2004) 292:331–7. 10.1001/jama.292.3.331 15265847

[91] Zeitler P Arslanian S Fu J Pinhas-Hamiel O Reinehr T Tandon N. ISPAD Clinical Practice Consensus Guidelines 2018: Type 2 diabetes mellitus in youth. Pediatr Diabetes. (2018) 19 Suppl 27:28–46. 10.1111/pedi.12719 29999228

[92] Libman IM Miller KM DiMeglio LA Bethin KE Katz ML Shah A. Effect of metformin added to insulin on glycemic control among overweight/obese adolescents with type 1 diabetes: a randomized clinical trial. JAMA. (2015) 314:2241–50. 10.1001/jama.2015.16174 26624824

[93] Bjornstad P Schafer M Truong U Cree-Green M Pyle L Baumgartner A. Metformin improves insulin sensitivity and vascular health in youth with type 1 diabetes mellitus. Circulation. (2018) 138:2895–907. 10.1161/CIRCULATIONAHA.118.035525 30566007 PMC6428045

[94] Anderson JJA Couper JJ Giles LC Leggett CE Gent R Coppin B. Effect of metformin on vascular function in children with type 1 diabetes: a 12-month randomized controlled trial. J Clin Endocrinol Metab. (2017) 102:4448–56. 10.1210/jc.2017-00781 29040598

